# A cell spot microarray method for production of high density siRNA transfection microarrays

**DOI:** 10.1186/1471-2164-12-162

**Published:** 2011-03-28

**Authors:** Juha K Rantala, Rami Mäkelä, Anna-Riina Aaltola, Petra Laasola, John-Patrick Mpindi, Matthias Nees, Petri Saviranta, Olli Kallioniemi

**Affiliations:** 1 Medical Biotechnology, VTT Technical Research Centre of Finland, 20521 Turku, Finland; 2 Institute for Molecular Medicine Finland (FIMM), University of Helsinki, 00014 Helsinki, Finland

## Abstract

**Background:**

High-throughput RNAi screening is widely applied in biological research, but remains expensive, infrastructure-intensive and conversion of many assays to HTS applications in microplate format is not feasible.

**Results:**

Here, we describe the optimization of a miniaturized cell spot microarray (CSMA) method, which facilitates utilization of the transfection microarray technique for disparate RNAi analyses. To promote rapid adaptation of the method, the concept has been tested with a panel of 92 adherent cell types, including primary human cells. We demonstrate the method in the systematic screening of 492 GPCR coding genes for impact on growth and survival of cultured human prostate cancer cells.

**Conclusions:**

The CSMA method facilitates reproducible preparation of highly parallel cell microarrays for large-scale gene knockdown analyses. This will be critical towards expanding the cell based functional genetic screens to include more RNAi constructs, allow combinatorial RNAi analyses, multi-parametric phenotypic readouts or comparative analysis of many different cell types.

## Background

High-throughput screening (HTS) of cellular effects of RNA interference libraries (siRNA or shRNAs) is now being increasingly applied to explore the role of genes in specific cell biological processes and disease states. However, the technology is still limited to specialty laboratories due to the requirements for robotic infrastructure, access to expensive reagent libraries, expertise in HTS assay development, standardization, data analysis and applications.

Currently, most described systemic-scale RNAi studies have been performed using microplate based screening platforms. Due to the high reagent demand of well based applications, these studies are often restricted to the analysis of a single parameter [1-3], cell morphology [4,5] or reporter gene assays [6-8] at a time. Though well suited for specific biological questions, this provides a restricted view on cell biology and furthermore, many informative assays cannot be converted to HTS format in microplate based platforms due to methodological limitations or cost considerations. In the future, a significant increase in the platform flexibility and decrease in screening costs will be required to expand functional large-scale screens to include more RNAi constructs, allow combinatorial siRNA analyses (e.g. gene-gene, gene-drug or gene-condition interaction), multi-parametric phenotypic readouts or comparative analysis of many different cell types. Such comprehensive perturbation of gene networks in cells will be critical towards understanding of cell and cancer biology as well as the discovery of novel therapeutic opportunities.

The transfection cell microarray technology has been proposed as a new platform for large-scale RNAi analyses allowing significant increase in experiment throughput and reduction in screening costs. The initial publication of transfection cell microarrays described reverse transfection of cDNAs to adherent cells [9]. Cells were grown as a uniform carpet over a printed microarray consisting of cDNA expression constructs and lipid transfection reagent resulting in the overexpression of the target genes in living cells. The concept was then adapted to RNAi analysis with description of cell carpet arrays for transfection of synthetic siRNAs to adherent cells [10,11]. After these primary publications, several groups have reported further development and application of different types of cell microarray methods [12-20]. For example, these methods have been used extensively to investigate cell cycle in human cancer cells [21]. Though the cell microarrays have slowly evolved to become a more widely accepted screening technology, in many publications, the individual arrays have contained only a modest number of samples, and data from multiple small arrays have been combined for large-scale coverage due to technological limitations of the methods [16,20,21].

Here, we describe the optimization of a cell spot microarray (CSMA) method with the future systemic-scale research needs in mind. The method provides a patterned array platform with spatially confined cell spots that allow simple production of cell microarrays with significantly increased sample coverage in microplate-sized array plates readily compatible with standard imaging instruments. The confined cell spot pattern also facilitates automated imaging and analysis of the arrays with multiparametric assays otherwise difficult or non-feasible in HTS. To allow rapid adaptation of the technique we optimized an application protocol of the CSMA cell patterning method for 85 cell types and applied the platform for functional genetics profiling of G-protein coupled receptor coding genes in cultured prostate cancer cells and non-malignant epithelial prostate cells, demonstrating the potential of the CSMAs for context specific target discovery.

## Results

### Cell spot microarray method

**Figure 1:**
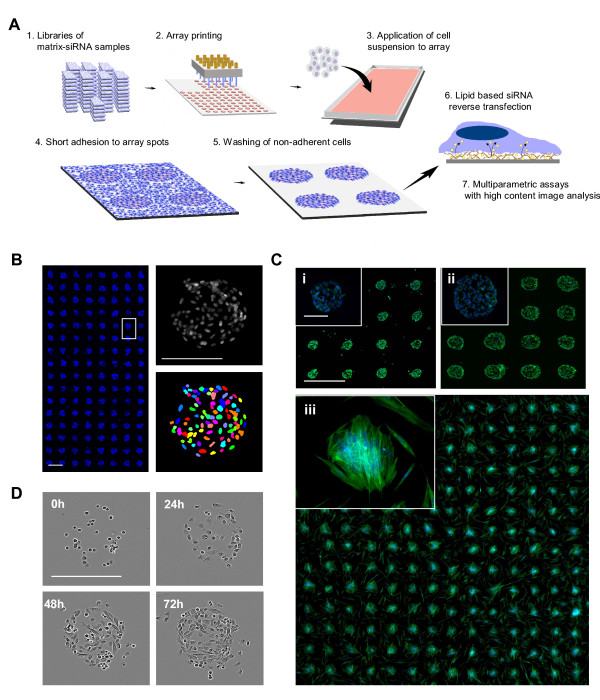
**Principle of the cell spot microarray (CSMA) method**. (A) CSMA work flow: siRNA samples in a printing solution containing transfection lipid and extra-cellular matrix proteins are robotically printed on a hydrophobic polystyrene surface. A suspension of adherent cells is allowed to adhere onto the array spots, followed by washing un-adhered cells off, leaving adhered cells only to printed array positions. After reverse transfection for a selected time, the arrays are stained using e.g. traditional multi-label immunostaining protocols for high content image analysis of multiple parameters. (B) CSMA method allows production of high density patterned cell arrays. Left panel displays a microarray scanned view of 200 μm cell spots with 500 μm spot spacing. Scale bar 0,5 mm. Right panel: Due to the spatially confined layout of the spots, automated imaging and segmentation of cells on CSMA spots can be performed using automated image analysis software. Scale bar 200 μm. (C) Microscopic image and microarray scanned view of 200 μm CSMA spots (Top left panel;i) and 400 μm spots (Top right;ii) of PC-3 cells stained for DNA (blue) and F-Actin (green) after 48 h culture. Microscopic image and microarray scanned view of 200 μm CSMA spots (Bottom;iii) of primary prostate stromal cells cultured for 48 h and stained for DNA (blue) and F-Actin (green). Scale bar 900 μm. (D) Phase contrast microscopic images from a timelapse series of PC-3 cells cultured on 200 μm array spots for 72 h.

In the CSMA method siRNA samples complexed with lipid transfection agent and extracellular matrix components are microarrayed with contact printing to a microplate sized array plate with a hydrophobic polystyrene surface (Figure 1A). Adherent cells are dispersed over the array as suspension and allowed to adhere for a short period, commonly 5 to 15 minutes before unadhered cells, unable to make permanent contact onto the array background during the initial adherence incubation, are washed off. After the wash step cells are left growing only on the spatially confined spots, providing a platform compatible for ultra-high sample densities and simplified imaging and quantification of cells on the siRNA spots (Figure 1B). As the patterning of adhesion promoting siRNA-matrix samples onto the arrays is done with contact printing the diameter of array spots and hence number of cells per spot can be controlled with printing pin diameter. Number of cells per spot is though dependent on the characteristics and growth properties of the cells. With epithelial derived PC-3 prostate cancer cells, number of cells on 200 μm spots following 48 h culture was 51 ± 3 (s.d., replicate spots n = 100) and 151 ± 8 on 400 μm spots. With primary un-immortalized prostate stromal cells, number of cells on 200 μm spots was 21 ± 6 (s.d., n = 192 spots) (Figure 1C). A microscopic 72 h timelapse imaging of PC-3 cells growing on a 200 μm CSMA spot (Figure 1D and Additional file 1) indicates how cells continue to prefer the printed matrix surface for their growth for several days.

**Figure 2:**
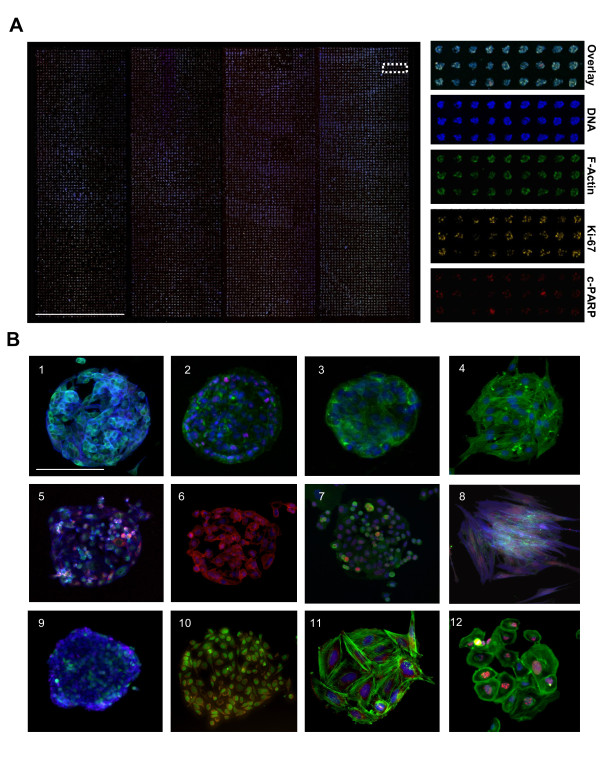
**Preparation of high density cell microarrays with multiple cell types using CSMA method**. (A) Laser microarray scanned composite image of VCaP prostate cancer cells cultured for 72 h on a four welled CSMA plate with 15,552 siRNAs spots (200 μm) printed as four sub-arrays with 3,888 spots in 18 mm × 54 mm areas. Magnified images of the array displaying the parallel staining of the cells for DNA, F-Actin, Ki-67 and cleaved PARP. Scale bar 18 mm. (B) Microscope images of 200 μm diameter array spots with: (1-12) VCaP, OVCAR-8, Caco-2, 3T3, PC-3, T-98G, KPL-4, primary prostate stromal cells, HEK-293, HeLa, SAOS-2 and primary osteosarcoma cells cultured on CSMAs for 48 h and stained with multiple types of immunostainings. Scale bar 100 μm.

Due to the high precision of contact microarray printing the method can be scaled for ultra-high sample densities. With 200 μm array spots and 500 μm spot spacing, arrays with 3,888 spots in an area of 18 × 54 mm or 15,552 spots in a single microplate-sized vessel with four large rectangular wells could be printed (Figure 2A) [22]. To achieve an array format compatible with majority of commonly used adherent cell types, different array surface materials were tested in combinations with different ECM protein coatings, cell dissociation procedures and adhesion incubation times for different cell types. Arraying of Matrigel ECM mixture onto a polystyrene surface with a hydrophobic surface charge was found to facilitate spatially restricted adhesion of majority of tested cell types (92%, 85/92) (Figure 2B). Although untreated polystyrene is not cell-repellent and cells are eventually able to adhere also to the array background, use of a non-enzymatic cell dissociation reagent and restricted cell adhesion time was found to significantly enhance spatial adherence of all tested cell types onto the array spots over the plain plastic background. With VCaP prostate cancer cells, the incubation time required for adhesion of cells to CSMA spots was shortened by 48-fold in comparison to the trypsin based cell dissociation technique (15 min vs. 12 h). Since the same array spot composition supported adhesion and growth of majority of tested cell types it was only necessary to optimize the cell dissociation method and adhesion time for each cell type [Additional file 2].

### siRNA reverse transfection on CSMAs

**Figure 3:**
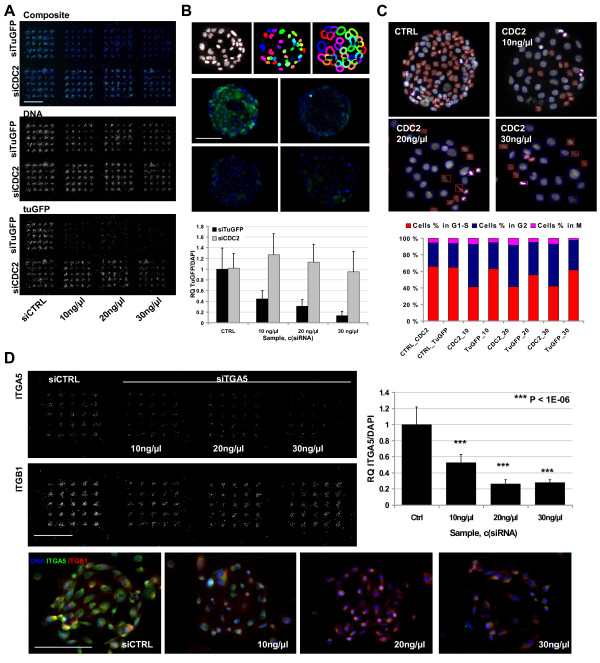
**siRNA reverse transfection on CSMAs**. (A) siRNA mediated gene silencing on CSMA was tested with U-2OS sarcoma cells expressing a destabilized TurboGFP. Silencing efficacy of different siRNA concentrations was compared with negative control siRNA and a control siRNA for CDC2. Composite (TuGFP = green, DNA = blue) and single channel confocal laser microarray scanned images of a U-2OS array with three 25 replicate spot sub-grids of siTuGFP and siCDC2 and two control siRNA sub-grids. Scale bar 2 mm. (B) Top: Automated image analysis was used to segment nuclei of individual cells and fixed area spanning 15 pixels outside nucleus was used to measure the cytoplasmic signal for TuGFP. Middle: 20× microscopic images of control siRNA (UL) and TuGFP siRNA spots (UR = 10 ng/μl, LL = 20 ng/μl, LR = 30 ng/μl). Quantification of cytoplasmic TuGFP against nuclear DAPI intensity indicated an up to 90% transfection efficacy with 30 ng/μl siRNA. Scale bar 100 μm. (C) Top: Image based cytometry analysis of integrated nuclear DAPI intensity (nuclear area/DAPI mean intensity) was used for cell cycle analysis of siCDC2 and siTuGFP transfected cells. Coloured boxes indicate cells classified as G1-S (red), G2 (Blue) and M (purple). Inhibition of CDC2 induced a prominent G2 arrest with all siRNA concentrations in comparison to control or TuGFP. (D) Top: Microarray scanned images of a CSMA with PC-3 cells stained for ITGA5 and ITGB1 following 48 h transfection with control and ITGA5 siRNA. Top right: Based on microscopic image analysis of ITGA5 signal against DAPI counterstaining of the cells, an up to 75% knockdown was achieved across the 25 replicates with 20 ng/μl ITGA5 siRNA. Bottom: 20× microscopic images of the control and ITGA5 siRNA spots. Scale bar 2 mm and 100 μm.

To establish lipid based siRNA reverse transfection on cell spot microarrays, we aimed for low siRNA and lipid concentrations in order to suppress potential lipid toxicity and off-target effects. Efficacy of siRNA transfection on CSMAs was tested by printing 200 μm array spots from 10-30 ng/μl siRNA samples [19]. Efficacy of target silencing was evaluated with U-2OS sarcoma cells expressing a destabilized TurboGFP (TuGFP). Cells were transfected for 72 h on an array with 25 print replicates of a validated TuGFP siRNA in three concentrations (Figure 3A). TuGFP signal intensity was analyzed using automated microscopic analysis. With normalization of the cytoplasmic TuGFP signal to the nuclear DNA counterstaining (Figure 3B) of the cells, an up to 60% efficacy was measured with the lowest 10 ng/μl siRNA concentration (P = 3.7E-06) across all replicate transfections, an over 70% efficiency was achieved with 20 ng/μl siRNA samples (P = 1.7E-08) and an up to 90% silencing efficacy was achieved with the 30 ng/μl siRNA samples (P = 1.2E-10). To evaluate specificity of the siRNA induced effects, an identical array of a validated siRNA for CDC2 (*CDK1*, cyclin-dependent kinase 1) was used as a control for the TuGFP analysis (Figure 3A). With comparative image based cytometry analysis of the siCDC2 transfected cells (Figure 3C) and the TuGFP transfected cells, the CDC2 inhibition was found to induce a prominent G2 cell cycle arrest with 51.1% of cells in G2 after 72 h transfection with the 10ng/μl siRNA (P = .0003), 49.8% with 20 ng/μl siRNA (P = 3.8E-05) and 50.6% with 30 ng/μl siRNA (P = 0.0006) in comparison to the 28.2% of cells in G2 in the control transfections (Figure 3C). Transfection with the TuGFP siRNA had an insignificant effect on the cell cycle distribution with all the siRNA concentration (Figure 3C).

Silencing of an endogenous target gene was evaluated with antibody based immunofluorescence detection of integrin alpha-5 (ITGA5) in PC-3 prostate cancer cells transfected for 48 h on a CSMA with replicate spots of a validated ITGA5 siRNA. Here, an up to 75% average silencing efficacy was measured from 25 replicate transfections with 30 ng/μl siRNA (P = 3.04E-08) (Figure 3D). Endogenous target silencing was further evaluated with antibody based detection of calpain 2 (CAPN2) silencing on VCaP prostate cancer cells transfected for 72 h on a CSMA with 96 technical replicates of four different *CAPN2 *and control siRNAs [Additional file 3: Supplemental Fig. S1A]. Based on confocal microarray laser scanning analysis of the cumulative spot level CAPN2 staining intensity against DNA counterstaining of the cells [Additional file 3: Supplemental Fig. S1B], a mean transfection efficacy of 31 to 94% silencing per siRNA construct was achieved in comparison to the negative control siRNA transfected cells, using 10 ng/μl siRNA concentration. For the most efficient CAPN2 siRNA (SI03057306), the inter-spot efficacy CV between all the replicate spots was 6.6%. To evaluate that the detected variation of the siRNA construct efficacy was independent of the transfection method, VCaP cells were transfected in conventional 96-wells for 72 h and analyzed with Western blot. An identical efficacy profile for the CAPN2 siRNA constructs was identified [Additional file 3: Supplemental Fig. S1C] thus highlighting the dependency of siRNA mediated gene silencing on the siRNA construct specificity. To further validate the transfection efficacy with VCaP cells we performed a Western blot analysis of cells transfected for 72 h on an array with 384 (200 μm) replicate spots of the most effective CAPN2 siRNA and an identical array of negative control siRNA. Here, an up to 85% silencing efficacy was achieved, verifying the immunofluorescence detected high transfection efficacy. In comparison, an identical assay using PC-3 prostate cancer cells and SVpgC2a immortalized primary oral keratinocyte cells (Kindly provided by Prof. Roland Grafström) indicated an up to 90 and 65% transfection efficacy (respectively) [Additional file 3: Supplemental Fig. S1D].

### Application of the CSMA method for systematic functional genetics analyses

**Figure 4:**
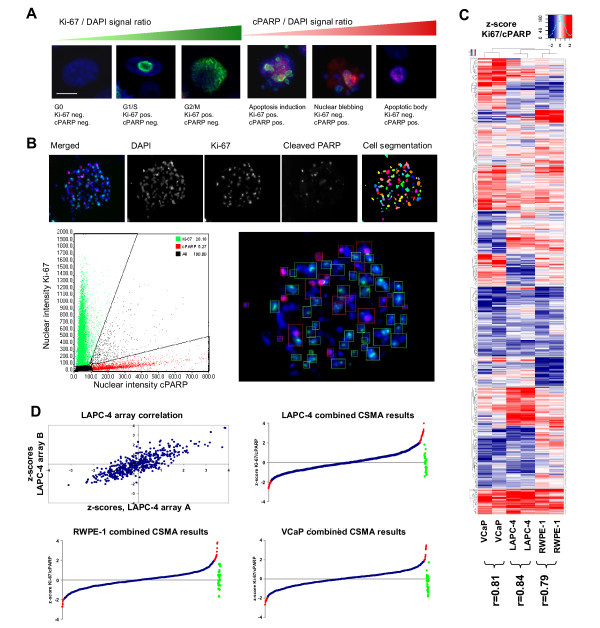
**Functional RNAi profiling of GPCR coding genes with prostate cell lines**. (A) An antibody based assay for detection of nuclear Ki-67 and cleaved PARP was used for microscopic analysis of cell proliferation and induction of apoptosis (blue = DNA, red = cPARP, green = Ki-67). In comparison to DNA counterstaining the nuclear intensity for Ki-67 staining increases linearly from G1 to M cell cycle phase whereas the staining for cPARP increases similarly from induction of apoptosis to formation of apoptotic bodies. (B) For the analysis each array position was imaged using x20 objective. Cells were segmented on basis of DAPI counterstaining and the intensities of Ki-67 and cPARP were measured from the nuclear area. On the whole array level the markers displayed a mutually exclusive staining pattern (Bottom left) allowing robust delineation of proliferating and apoptotic cells (Bottom right). The green and red rectangles on the image display the gated populations of Ki-67 or cPARP positive cells respectively. (C) Hierarchical partitioning around medoids (PAM) clustering of the standardized (z-score) results of the two biological replicate CSMA experiments with LAPC-4, RWPE-1 and VCaP cells. (D) Top left: Scatter plot distribution of the replicate experiment z-score results for LAPC-4 cells. Top right and bottom: Line graph distribution of the combined replicate experiment results for each siRNA construct for the analyzed cell lines. SiRNAs inducing a greater than ± 2 z-score (displayed in red) were considered significant. Distribution of the negative control siRNAs shown in green.

To validate the compatibility of the CSMA method in systematic RNAi screening we established an assay for analysis of G-protein coupled receptor (GPCR) coding genes impacting on the growth and survival of cultured prostate cells. A siRNA library with two siRNA constructs against 492 human GPCRs and replicate negative control siRNA samples was used for preparation of arrays used for parallel analysis of GPCRs important for growth of androgen responsive VCaP and LAPC-4 prostate cancer cells and RWPE-1 non-malignant prostate epithelial cells. To allow microscopic detection of RNAi effects impacting cell proliferation and survival, we established an antibody based assay for detection of Ki-67 as a proliferation marker and cleaved PARP (poly (ADP-ribose) polymerase) as an apoptosis marker. The markers show a mutually exclusive staining pattern allowing simple phenotypic delineation of proliferating and apoptotic cells on basis of the nuclear staining intensity of the proteins against DNA counterstaining of the cells (Figure 4A &4B). The Ki-67 protein is detected abundantly in the nucleus surrounding the chromatin in all phases of active cell cycle with an increasing intensity from G1 to M, whereas PARP is cleaved in response to induction of apoptosis resulting in increasing cPARP level from the early events of apoptosis induction to formation of apoptotic bodies (Figure 4A).

For the screening two identical arrays with random order printed individual siRNA samples were used to transfect each cell type for 48 h followed with immunofluorescent staining of the cells for analysis using automated microscopic imaging (Olympus scanR). Each array position was imaged using a long aperture LUCPLFLN x20 objective and automated image analysis software (Olympus scanR) was used to segment the cells and analyze the nuclear staining intensities of the two antibodies (Figure 4B). The spot level ratio of RNAi induced changes in the cumulative nuclear intensities of Ki-67 and cPARP was used to identify target genes causing inhibition of cell proliferation and induction of apoptosis upon silencing. By comparison of the scored results of the two replicate arrays for each cell type, the CSMA experiments displayed a statistically significant concordance with Pearson correlations between r = 0.79 to 0.84 (Figure 4C &4D). For stringent identification of genes having the most consistent impact on growth and survival of the analyzed cell lines, results of the replicate experiments were combined and siRNAs having a z-score greater than ± 2 s.d. for the measured Ki-67/cPARP signal ratio over both experiments were considered significant (Figure 4D and Additional file 4).

### *In silico *transcriptomics validation

**Figure 5:**
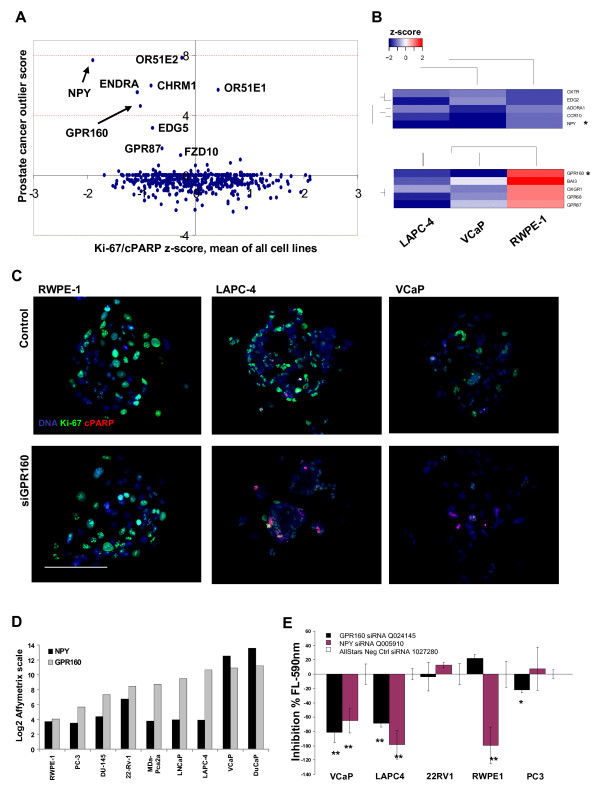
**Validation of CSMA screening results**. (A) Comparison of the mean z-score of Ki-67/cPARP index of all analyzed cell lines with clinical gene expression levels of GPCRs in clinical prostate cancer samples (n = 349) vs. healthy prostate samples (n = 147) http://www.genesapiens.org. (B) Unsupervised hierarchical clustering of the averaged siRNA z-scores for the top 5 highest ranking candidate genes identified on basis of rank product analysis of common impact on all the cell types (Top) and on basis of prostate cancer cell line specific growth inhibitory impact (Bottom). (C) Representative x20 microscope images of the GPR160 (siRNA Q024145) CSMA spots for RWPE-1, LAPC-4 and VCaP cells. Scale bar100 μm. (D) Comparison of mRNA transcript levels of GPR160 and NPY in a panel of commonly used prostate model cell lines. Log2 Affymetrix scale. (E) An enzymatic cell viability assay was used to verify the Ki-67/cPARP based growth inhibitory effect of GPR160 and NPY in VCaP, LAPC-4, 22Rv1, RWPE-1 and PC3 cells. ** p < .01, * p < .05.

To address the *in vivo *significance of the highlighted GPCRs in clinical prostate cancer samples, we carried out a bioinformatic meta-analysis of the clinical expression profiles of the corresponding genes at the mRNA level from the publicly available GeneSapiens gene expression database (http://www.genesapiens.org, [23]). To evaluate potential prostate specificity of the analyzed GPCRs, we first compared the median expression levels of the genes in healthy prostate (n = 147) and prostate cancer samples (n = 349) with the normalized expression levels of the genes across over 10,000 clinically annotated tissue samples from other major anatomical tissue types. Here, seven genes; *OPRK1*, *OR51E1*, *OR51E2*, *GPR160*, *NPY*, *CHRM1 *and *EDG7 *displayed increased expression in prostatic samples in comparison to other tissue types [Additional file 5]. To identify genes displaying outlier expression patterns in clinical prostate cancer samples we calculated an outlier expression score [24] for the genes by comparing the median expression levels of the genes in prostate cancer samples over the healthy prostate control samples [Additional file 6]. Out of the 492 GPCRs analyzed in the CSMA experiment, 9 were identified to display an increased expression pattern in the prostate cancer samples (Figure 5A). To identify correlations between the *in vivo *prostate cancer specificity and the loss-of-function impact of the genes on the prostate cell lines, the outlier expression scores were combined with the summarized CSMA results for the corresponding genes. *NPY *(neuropeptide Y) and *GPR160 *(G protein-coupled receptor 160) [Additional file 7] were among GPCRs with the highest prostatic tissue level and prostate cancer specificity index. With analysis of the CSMA results on basis of rank product analysis [25] of all the three cell lines, NPY was identified as the strongest common growth inhibitory siRNA hit in all the three cell lines (Figure 5B), while GPR160 had the strongest growth inhibitory effect on the cancer cell lines VCaP and LAPC-4, and an insignificant effect on the RWPE-1 epithelial cells (Figure 5B-5C), coupling with its overexpression in clinical prostate cancer samples in the Genesapiens database analysis (p = 3.10E-05, t-Test -5.749) [Additional file 5]. High level *in vivo *expression of the GPR160 protein was also identified in clinical prostate cancer specimens in a public tissue protein profiling database (http://www.proteinatlas.org, [26]) [Additional file 8: Supplemental Fig. S4A] and a separate previously published transcriptomics analysis [27] of 40 human prostate cancer samples (p = 2.22E-04, fold change 3.246).

### Validation of the screening results

To validate the growth inhibitory knockdown effect of the two highlighted GPCR coding genes (NPY and GPR160) a series of secondary validation experiments were performed. The efficacy of the GPR160 and NPY siRNAs was validated at the mRNA level in VCaP cells by means of quantitative RT-PCR [Additional file 8: Supplemental Fig. S4B]. By comparison of the mRNA expression level of GPR160 and NPY in the analyzed cell lines and six additional prostate cancer cell lines http://www.genesapiens.org, VCaP cells were identified with the highest expression of both two genes in comparison to LAPC-4 and RWPE-1 cells (Figure 5D). To validate the growth inhibitory effect detected in the CSMA analysis, we used an enzyme activity based fluorometric cell viability assay and a panel of prostate cancer cell lines to verify the effect. Five prostate cell lines; VCaP, 22Rv1, LAPC-4, PC-3 and RWPE-1 were transfected in conventional 96-wells for 72 h and assayed with a fluorescence plate reader. A significant reduction of cell viability (p < .01) by inhibition of NPY was measured for all three cell lines included in the CSMA analysis. Silencing of GPR160 was found to decrease viability of VCaP and LAPC-4 (p < .01) cells and moderately the viability of PC-3 prostate cancer cells (p < .05), but not RWPE-1 (Figure 5E), thus validating the rank product analysis based CSMA results. Even though siRNAs for NPY and GPR160 did not exceed the used -2 s.d. threshold in the primary CSMA analysis of LAPC-4 cells, they both scored in the validation assay. A possible reason for the difference in the results is the difference of the two assay readouts. The antibody based readout potentially reflect also the apoptotic mechanism, whereas the fluorometric assay is dependent solely on the number of viable cells independent of underlying mechanisms resulting in the cell killing. By viewing the resulting phenotype of LAPC-4 cells (Figure 5C) following GPR160 silencing, a residual staining for Ki-67 is detected along with an increased staining for cPARP thus resulting in a smaller signal ratio than with e.g. the distinct decreased Ki-67 and increased cPARP signal ratio in VCaPs (Figure 5C). It is also possible that in the CSMA format apoptotic cells become detached from the array spots before the staining thus not scoring in this analysis setting.

## Discussion

We describe here the optimization and functional application of a miniaturized cell spot microarray (CSMA) method for RNAi screening. The technique has several advantages as compared to conventional HTS screening in microplate format and enhancements also applicable to previously described cell microarray technologies. Most importantly, miniaturized high density transfection microarrays provide an affordable platform for large-scale cell biological studies. The described CSMAs provide a method for production of transfection cell arrays with sample densities allowing screening of up to 15,000 siRNA molecules in a single array plate. Consumption of the siRNAs and detection reagents in the CSMA technique is over 200-fold less than with traditional HTS in 384-well plates (10 ng siRNA/well vs. 50 pg/spot). Immunofluorescent staining of CSMAs can be accomplished under a coverslip reducing antibody consumption up to 400-fold as compared to 384-well format (10 μl antibody dilution per 384 well vs. 320 μl per array plate with 15,552 siRNA samples). Therefore, it is possible to develop sophisticated HTS assays that would otherwise not be feasible, and easily convert almost any cytochemical staining to a HTS assay.

As compared to most of the cell microarray methods, where a carpet of cells is laid across the whole array [9-12,14-17,19-21], the CSMA method provides a simplified protocol for achieving a patterned array layout, where cells only adhere to the sample spots. This configuration facilitates the quality control and digital image analysis of the cell arrays as cells included in the analysis can be defined without possibility for sample mixing between array positions. With future development of higher capacity timelapse microscopic imagers, this platform could also be used for high-throughput timelapse microscopic analyses.

Since in the method cells are seeded over the entire array at a time and the spot diameter is limiting the number of cells adhering to the spots, well-to-well variability inherent in classical HTS systems is reduced in CSMAs, potentially increasing the accuracy of the screens. Furthermore, the arrays are simple to produce and applicable to various commercially available polystyrene cell culture vessel formats and moreover scalable, limited only by the microarray printing capacity and throughput of the image analysis systems. The confined cell spot arrays were accomplished by including in the printing mixture matrix proteins, which were deposited on a standard untreated hydrophobic polystyrene surface. In addition to promoting cell adhesion, the ECM matrix proteins have been shown to enhance lipid based reverse transfection through promotion of cell spreading and activation of endocytotic pathways in adherent cells, hence yielding an enhanced transfection efficacy [28-30]. 92 different cell types, including human primary cells, were tested with the approach and an application protocol was optimized for 85 cell lines which could be successfully seeded and cultured on the arrays.

## Conclusions

The biological application utilized here to demonstrate the use of CSMAs for systematic RNAi screening, was the functional analysis for GPCR coding genes impacting growth and survival of prostate epithelial and prostate cancer cells. With the analysis we demonstrated that the CSMAs allow robust functional analysis of large siRNA sets using multiplexed assays, such as the antibody based assays and different cell types. The targets identified based on the integrated analysis of CSMA data and publicly available transcriptomics data displayed high context specificity suggesting GPR160 and NPY likely to be the two most important GPCRs impacting on growth of human prostate and specifically prostate cancer cells. While these genes have been previously associated with prostate cancer [31,32], this is the first comparative functional screening of the GPCR knockdown phenotypes in different cultured prostate cell lines, combined with the bioinformatic analysis of their relative expression levels in prostate tumor samples. Furthermore, several genes including *FZD4*, *OPRK1 *and *OXTR *identified here with a growth inhibitory loss-of-function impact on the prostate cancer cells have been recently associated in independent studies with a role in prostate cancer pathogenesis [33-35]. This illustrates the use of CSMA method in the integrated functional genomics analysis of *in vitro *RNAi data and *in vivo *clinical gene expression data, to quickly nominate biologically and clinically relevant candidate genes and potential drug targets for further analysis.

## Methods

### CSMA printing

The matrix-siRNA samples used for array printing were prepared to U-bottom 384 wells (Ab1055, Abgene) with a Hamilton STAR 96-channel liquid handling robot (Hamilton Robotics) in sterile conditions. For each sample 5 μl of 1.67 μM (final 10 ng/μl), 3.35 μM (20 ng/μl) or 5 μM (30 ng/μl) siRNA (Qiagen) stock diluted in OptiMEM-I (Gibco) was mixed with 0.8 μl, 1 μl or 1.2 μl (respectively) of siLentFect (Bio-Rad) transfection agent and 0.2 μl of OptiMEM I. Solutions were incubated for 25 min at RT and mixed with 2 μl of growth factor reduced Matrigel (BD Biosciences) and 2 μl of ice cold OptiMEM-I supplemented with 65 mM of sucrose, mixed by vortex shaking, centrifuged at 2.000 rpm for 1 min and snap-frozen at -80°C. Source plates were stored at -20°C between uses. Microarray printing was performed with a Genetix Qarray2 (Genetix Ltd) microarray printer using 16 solid tip pins with 200 μm tip (PointTechnologies). Arrays were printed onto clear untreated polystyrene microplates with four rectangular wells (Nunc). Printing was done in controlled 55% air humidity and source plates were kept at +15°C during printing. To minimize sample carry-over during the multi-sample print processes a five step distilled water rinsing (3 × 2.5 seconds and 2 × 5 s) of printing pins was used followed with 80% ethanol and air drying. Printed array plates were sealed in airtight bags and stored at room temperature before use.

### siRNA libraries and plasmids

All siRNAs used for the experiments were purchased from Qiagen. CSMA arrays used for GPCR analysis covered a library with 2 individual constructs against 492 human GPCR coding genes (GPCR subset of Qiagen human genome-wide siRNA library v1.0). All arrays used for the experiments had a printed subset of replicate negative control siRNA positions equivalent to 5% of all targeting siRNAs on the array (All star negative control, Qiagen 1027280). SiRNA identifiers (Qiagen) for CAPN2, GPR160 and NPY are provided in Figure 3 and 5, TuGFP siRNA (1027020), CDC2 (1027273) and ITGA5 (SI02654841). Plasmid for expression of TurboGFP in U-2OS cells was purchased from Evrogen (pTurboGFP-dest1 vector, cat.# FP519).

### Cell culture

All cell lines used for the testing of the CSMA method were cultured according to the protocols recommended for the cell line. U-2OS, VCaP, LAPC-4 and 22RV1 cells (ATCC, Manassos, USA) were grown in RPMI-1640 (Gibco) supplemented with 10% FBS, 10 μg/ml penicillin and streptomycin and 2 mM L-glutamine. RWPE-1 (ATCC, Manassos, USA) cells were grown in Keratinocyte Serum Free Medium (K-SFM, Gibco) supplemented with 0.05 mg/ml BPE (bovine pituitary extract) and 5 ng/ml EGF. PC-3 cells (ATCC, Manassos, USA) were grown in F-12K medium (Gibco) supplemented with 10% FBS, 10 μg/ml penicillin and streptomycin, and 2 mM L-glutamine. Turbo-GFP expressing U-2OS cells were established using Fugene HD (Promega) transfections reagent according to the instructions provided by the manufacturer. Cells were kept under G418 (Sigma-Aldrich) selection for 6 passages prior to assays.

### Preparing cells for CSMAs

To allow maximally confined growth of cells on the CSMA spots, specific cell dissociation and array adhesion protocols were developed. The protocol for VCaP cells can be used as a guideline for all other tested cell types. For the CSMA experiments, VCaP cells were grown to 80% confluence on 10 cm culture dishes and dissociated with HyQtase (HyClone) treatment for 5 minutes. Conditioned culture medium was collected from the dish and after dissociation; cells were re-suspended back to this and pipetted thoroughly to achieve a uniform single cell suspension. For experiments on the four well array plates, 2.5 × 10^6 cells in 4.5 ml of medium was added carefully to any corner of a single well, while avoiding application of cells directly onto the printed array surface and dispersed uniformly over the whole array surface. Cells were allowed to adhere to the arrays at +37°C, 5% CO2 for 15 min after which excess un-adhered cells were washed off by gently rinsing the arrays with PBS until all un-adhered cells were removed. For the transfection, 4.5 ml of fresh normal culture medium was added to array wells.

### Immunofluorescence staining

Immunofluorescence staining of the arrays was performed using standard procedures. Cells were fixed with 2% paraformaldehyde solution for 15 min, permeabilized with 0.3% Triton-X100 in PBS and the background was blocked with 2% BSA in PBS before staining with primary and secondary antibodies. Primary antibodies for ITGA5 (1:200, rabbit anti-ITGA5 clone 1949, Millipore), CAPN2 (1:250, rabbit anti-CAPN2, Abcam), cPARP (1:300, mouse anti-cPARP, Cell Signalling Technologies) and Ki-67 (1:300, rabbit anti-Ki67, Abcam) were diluted in blocking buffer and incubated for 60 min at room temperature, 80 μl per array under a coverslip. Secondary labelling antibodies; goat-anti-mouse and donkey-anti-rabbit conjugated with Alexa488 and -647 dyes (1:300, Molecular Probes, Invitrogen) were diluted in blocking buffer and incubated for 60 min at RT. 1 μg/ml 4',6-Diamidino-2-phenylindole (DAPI, Invitrogen) was added to secondary antibody solution for DNA staining. After secondary labelling, CSMAs plates were rinsed with distilled H2O, air dried and stored at room temperature protected from light before imaging.

### Microscopic imaging and data analysis

CSMA analysis was performed with microsopic imaging of the arrays using scanR high content imager (Olympus) equipped with a Hamamatsu ORCA-ER CCD digital camera (Hamamatsu Photonics K.K.). Each array spot was imaged individually with a x20, LUCPLFLN NA 0.40 objective using specific filter sets for DAPI, Alexa488 and Alexa647 (Semrock, Inc). Acquired images were analyzed using the scanR image analysis software. Cells on spots were segmented on the basis of the DNA counterstaining (Figure 1B) and the nuclear fluorescence intensities for DAPI, Ki-67 and cPARP were measured. Cells residing outside the main spot perimeter were excluded from the analysis on basis of x-y coordinates from the center of spot. Raw intensities of stains for all cells per spot were spatially normalized using pin normalization. Ki-67 and cPARP values were then used to calculate a Ki-67/cPARP ratio normalized against the DAPI counterstaining per spot. After normalization, a z-score was calculated for scoring of the measured spot level values using global array mean and standard deviation within all samples in the array. From the analysis, siRNAs with z-scores less than -2 s.d. or greater than +2 s.d. were considered significant and the consistency of the siRNA effects across all measurements or alternative impact on the cancerous cell lines (VCaP and LAPC-4) in comparison to the epithelial cells (RWPE-1) was performed using the rank product approach [25]. The partitioning around medoids method and the R statistical programming language was employed for hierarchical clustering of the data.

### Meta-analysis of prostate cancer gene expression datasets

The analysis of *in vivo *significance of the identified siRNA targets was based on analysis of public Affymetrix transcriptome data of prostate samples derived from the GeneSapiens database [23] using the GTI method as previously described [24]. The included data was acquired from multiple public repositories such as 1. The Gene Expression Omnibus (GEO); GSE1133, GSE1431, GSE2109, GSE2361, GSE2443, GSE3325, GSE3526, GSE5258, GSE6606, GSE6608, GSE6919 and GSE96. 2. The Human Genome Expression Index (HuGEIndex); HIE01. 3. The Broad Institute Cancer Program Data Sets; MTE05, MTE10, MTE11 and 4. The NIH-NCI Gene Expression Data Portal (GEDP); NCE279.

### Quantitative RT-PCR and Western blot analysis

For quantitative reverse transcription-PCR (qRT-PCR) analysis the total cellular RNA was isolated using Trizol reagent (Invitrogen). For cDNA synthesis, 200 ng of total RNA was reverse transcribed with the High Capacity cDNA Reverse Transcription kit (Applied Biosystems). The cDNA was diluted 1/10 and the Taqman qRT-PCR analysis was performed with an Applied Biosystems 7900HT instrument, using primers designed by the Universal Probe Library Assay Design Center (Roche) for NPY; forward CGCTGCGACACTACATCAAC and reverse CTCTGGGCTGGATCGTTTT, and for GPR160; forward GCATTCAGAGTTACTGGCTGTC and reverse CCCAACAGGTTATGAAAGCTACA. The fluorescent Taqman probes were obtained from Roche Human Probe Library. Results were analyzed using SDS 2.3 and RQ manager software (Applied Biosystems), and the relative expression of mRNA was determined using beta-actin (forward CCAACCGCGAGAAGATGA, reverse CCAGAGGCGTACAGGGATAG) as an endogenous control. The data from two separate biological experiments, with triplicate samples was combined. For western blot analysis aliquots of total cell lysates were fractionated on SDS-polyacrylamide gels and transferred to nitrocellulose membrane (Whatman Inc). The filters were blocked against non-specific binding using 5% skim milk. Membranes were probed with antibodies o/n at +4°C (CAPN2; 1:1000, Abcam). Equal loading was confirmed by probing the same filter with a specific antibody for tubulin (1:5000, Abcam). Signals were revealed by incubating the filters with horseradish peroxidase-coupled goat anti-mouse IgG secondary antibody and goat anti-rabbit (1:1000; Sigma) antibody.

### Conventional siRNA transfections and cell viability assay

Validation of target silencing and assaying the effect of NPY and GPR160 siRNAs on the growth of prostate cancer cells was performed in clear-bottom 96-well (2000 cell per well) and 24-well (5 × 10^4 cells per well) plates. The transfections were made with 10 nM siRNA constructs (Qiagen) using siLentFect transfection agent (Bio-Rad). Cell viability was assayed with CellTiter-Blue (CTB) fluorometric cell viability assay (Promega). 10 μl of CTB diluted with 10 μl of OptiMEM 1 (Gibco) medium without supplements was added to each 96-well containing 100 μl of medium and cells. Reagent was incubated at +37°C for 4 h followed with 2 h stabilization at RT before analysis. Fluorescence signal (Exitation 560 nm, Emission 590 nm) reflecting the relative number of viable cells per well was measured with EnVision fluorescence plate reader (PerkinElmer). Data of four replicate wells was combined for analysis.

## Competing interests

The authors declare that they have no competing interests.

## Authors' contributions

JR developed the CSMA method and performed the experiments. JR, OK and MN designed the experiments. JR, RM and PS performed the array printing and siRNA library formatting. ARA and PL performed the validation experiments. JPM performed the GTI transcriptomics analysis. JR and OK wrote the paper. The manuscript has been reviewed by the authors who all agree to its publication in BMC Genomics.

## Supplementary Material

Additional file 1 **Supplemental video 1**. Timelapse microscopic imaging of PC3 prostate cancer cells seeded on array with 200 μm spots and imaged for 72 h. Images were collected using a x20 objective with 30 minute intervals. Click here for file

Additional file 2 **Supplemental Table 1**. List of cell lines tested with the CSMA method. 92 adherent cell types were tested for compatibility with the CSMA cell patterning method. Four different cell dissociation methods were tested in parallel and protocol allowing maximally efficient cell patterning was optimized for all tested cell types. Click here for file

Additional file 3 **Supplemental figure S1**. (A) Validation of CSMA transfection efficacy in VCaP cells was performed using four different siRNA constructs for *CAPN2 *and a control siRNA. Composite (CAPN2 = red, DNA = blue) and single channel confocal laser microarray scanned images of a VCaP array with four 24 replicate spot sub-grids of three CAPN2 and control siRNA constructs. Scale bar 900 μm. (B) 20× microscopic images of control siRNA and CAPN2 siRNA spots with an intensity surface plot visualization of the intra-spot signal distribution of anti-CAPN2 staining (lower panels). (C) Upper panel: On basis of the immunofluorescent detection of CAPN2 an up to 94% spot level efficacy was measured with spot level normalization against DNA counterstaining of the cells. The mean efficacy of CAPN2 siRNAs varied from 31% to 94% silencing. Lower panel: Comparative Western blot analysis of cells transfected using conventional methods provided identical efficacy profiles for the siRNA constructs. (D) Western blot of analysis of cells recovered from a CSMA array of 384 replicate CAPN2 and control siRNA spots after 72 h transfection was used to validate the transfection efficacy in VCaP and PC-3 prostate cancer cells and SVpgC2a oral keratinocytes. Click here for file

Additional file 4 **Supplemental data 1**. Results summary table listing the CSMA results of the functional analysis of the GPCR targeting siRNAs. Click here for file

Additional file 5 **Supplemental figure S2**. Tissue level unsupervised hierarchical clustering of the 7 candidate primary RNAi hit genes identified with an increased expression pattern in clinical prostate samples. Each cell in the cluster shows the log2 expression ratio for the particular gene in separate tissue samples divided by the median expression of that gene in all the samples. Red; expression above the median, blue; below the median. Click here for file

Additional file 6 **Supplemental data 2**. Table listing the transcriptomics analysis results of the analysed genes in the Genesapiens transcriptomics database. Genes represented with a minimum of 10 analyzed healthy and cancer tissue samples were included. Click here for file

Additional file 7 **Supplemental figure S3**. Box plot graphs displaying normalized expression levels of GPR160 and NPY across major tissue groups divided into healthy and cancer samples in the Genesapiens transcriptomics database. Click here for file

Additional file 8 **Supplemental figure S4**. (A) Representative immunohistochemistry staining of tissue microarray cores of healthy and prostate cancer samples for GPR160 http://www.proteinatlas.org. (B) Knockdown performance of siRNAs targeting GPR160 and NPY. Transcript levels for duplicate experiments with triplicate samples were measured by quantitative RT-PCR and are reported for each siRNA in the increasing efficacy order relative to average transcript levels for negative control siRNA transfections. Click here for file
