# Supplementary figures and images for: A cell spot microarray method for production of high density siRNA transfection microarrays
Source: BMC Genomics. 2011 Mar 28;12:162. doi: 10.1186/1471-2164-12-162 (PMC3073923; doi:10.1186/1471-2164-12-162)

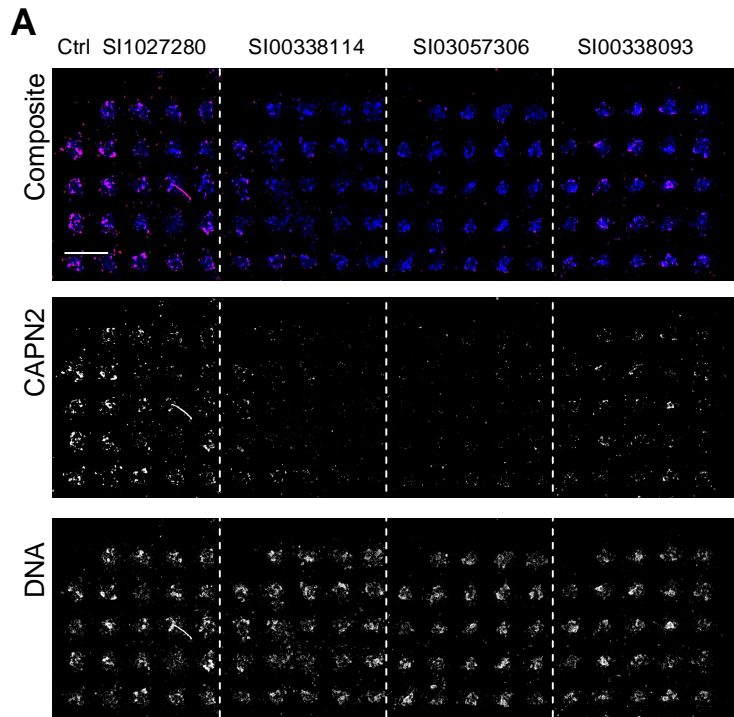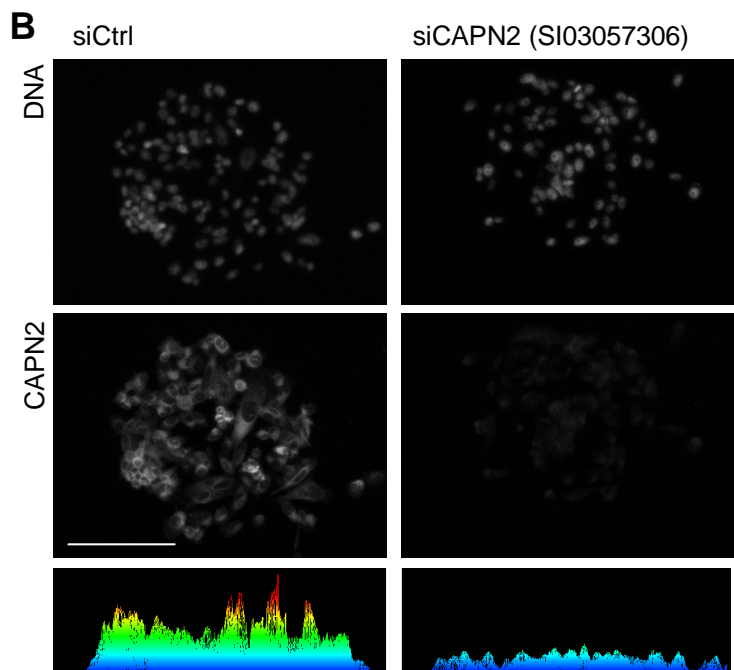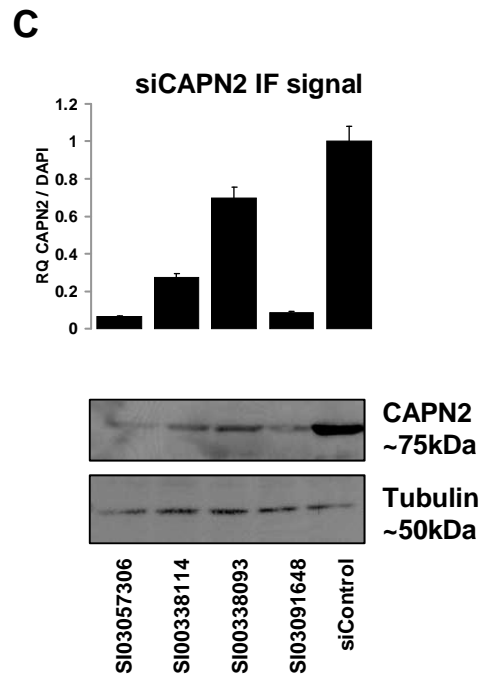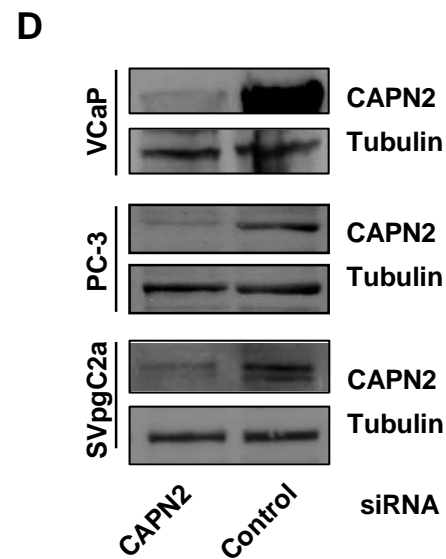

Supplement: Additional file 3 — Supplemental figure S1. (A) Validation of CSMA transfection efficacy in VCaP cells was performed using four different siRNA constructs for CAPN2 and a control siRNA. Composite (CAPN2 = red, DNA = blue) and single channel confocal laser microarray scanned images of a VCaP array with four 24 replicate spot sub-grids of three CAPN2 and control siRNA constructs. Scale bar 900 μm. (B) 20× microscopic images of control siRNA and CAPN2 siRNA spots with an intensity surface plot visualization of the intra-spot signal distribution of anti-CAPN2 staining (lower panels). (C) Upper panel: On basis of the immunofluorescent detection of CAPN2 an up to 94% spot level efficacy was measured with spot level normalization against DNA counterstaining of the cells. The mean efficacy of CAPN2 siRNAs varied from 31% to 94% silencing. Lower panel: Comparative Western blot analysis of cells transfected using conventional methods provided identical efficacy profiles for the siRNA constructs. (D) Western blot of analysis of cells recovered from a CSMA array of 384 replicate CAPN2 and control siRNA spots after 72 h transfection was used to validate the transfection efficacy in VCaP and PC-3 prostate cancer cells and SVpgC2a oral keratinocytes. [file 1471-2164-12-162-S3.PDF]

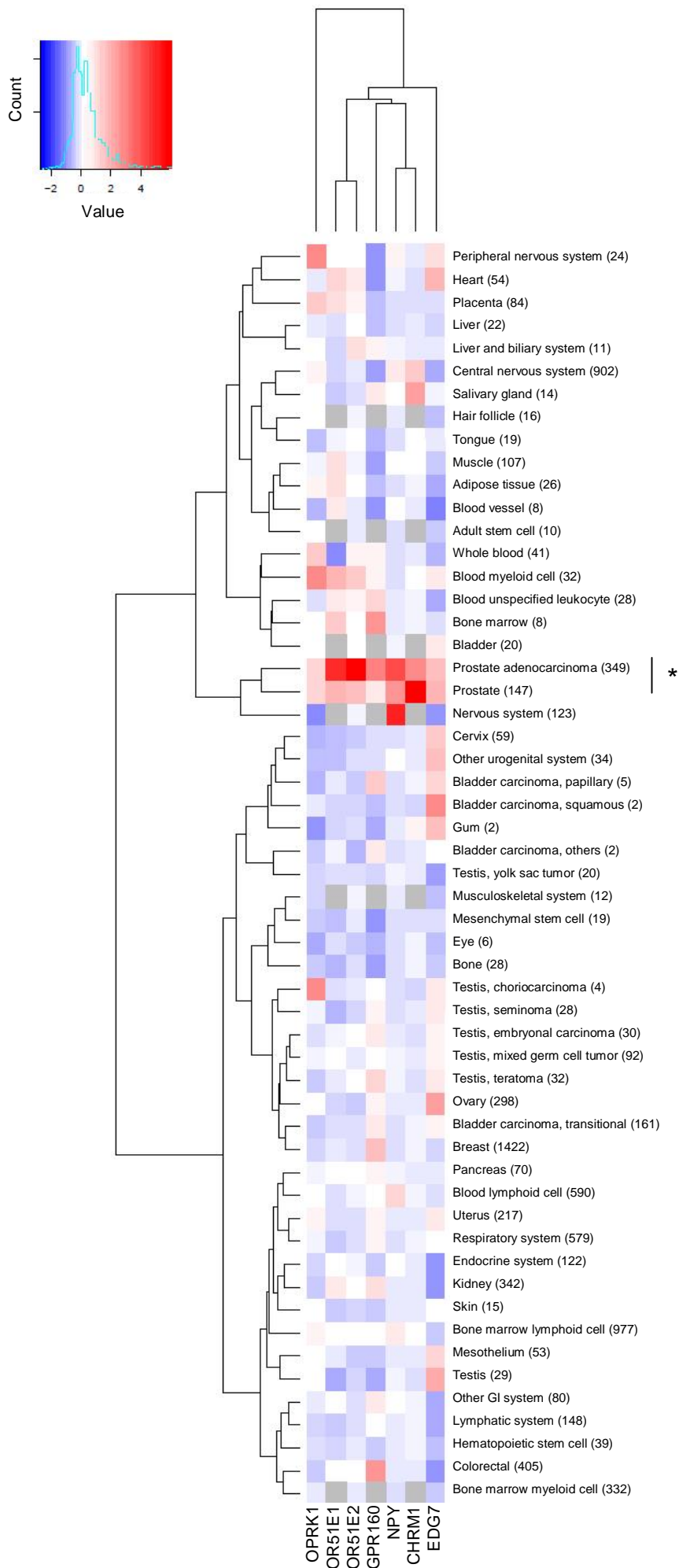

Supplement: Additional file 5 — Supplemental figure S2. Tissue level unsupervised hierarchical clustering of the 7 candidate primary RNAi hit genes identified with an increased expression pattern in clinical prostate samples. Each cell in the cluster shows the log2 expression ratio for the particular gene in separate tissue samples divided by the median expression of that gene in all the samples. Red; expression above the median, blue; below the median. [file 1471-2164-12-162-S5.PDF]

**GPR160, ENSG00000173890, normal vs cancer; t-test= -5.749442677, p=3.10E-05, n=75**

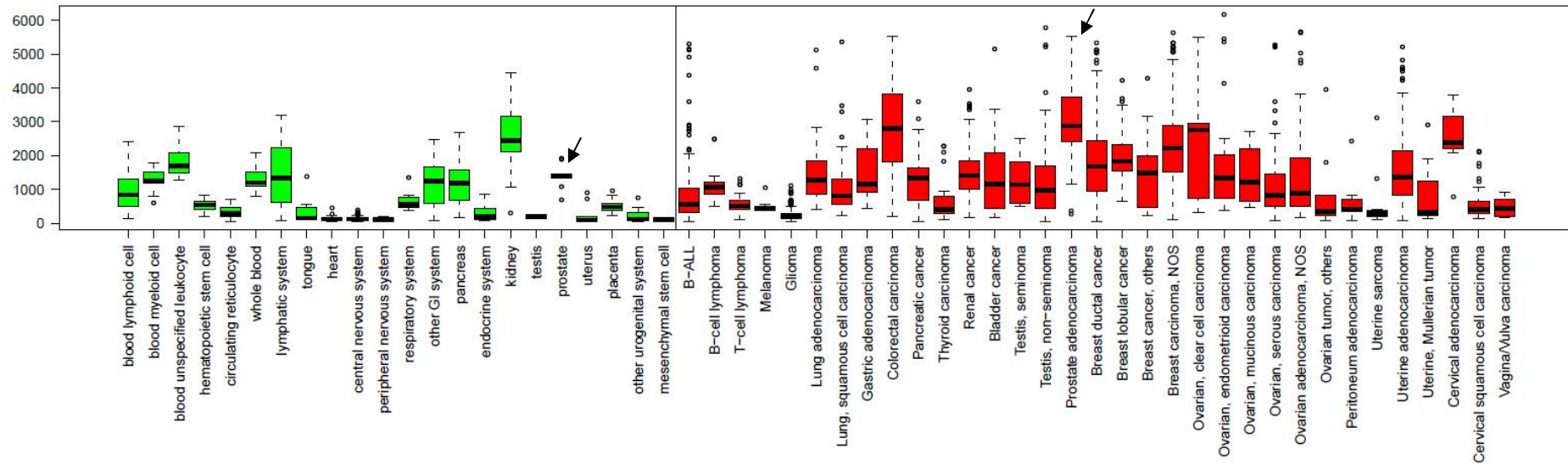

**NPY, ENSG00000122585, normal vs cancer; t-test= -3.434664327, p=0.00068, n=476**

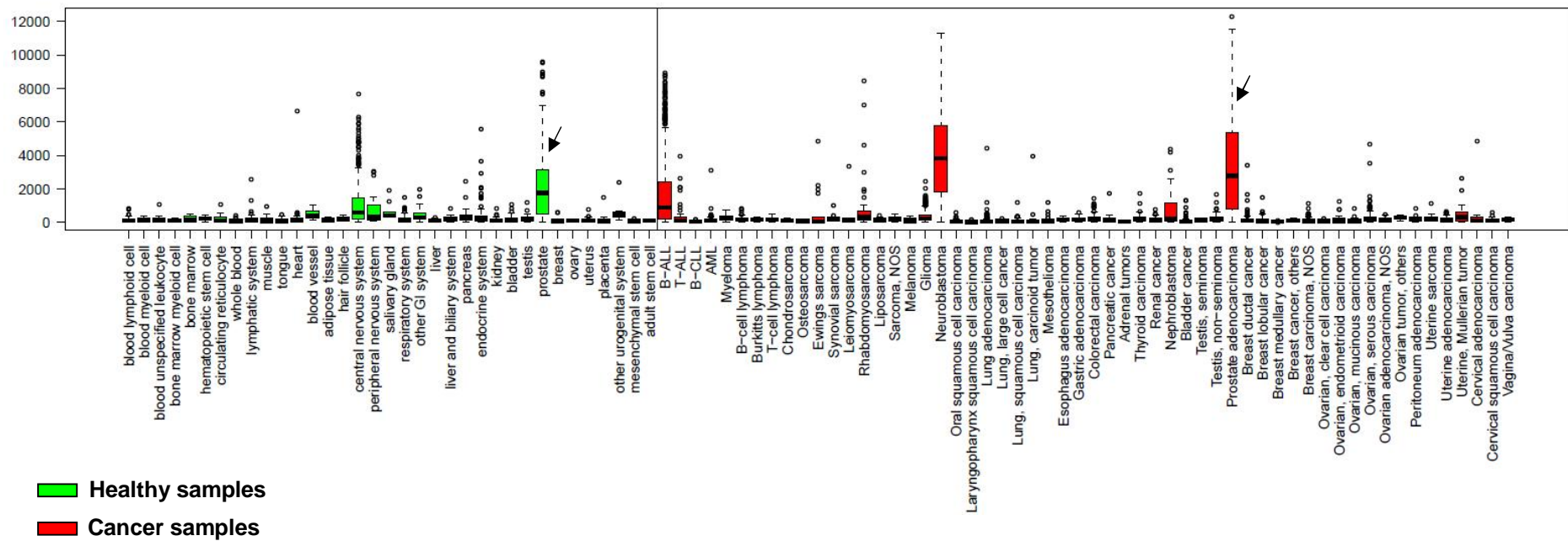

Supplement: Additional file 7 — Supplemental figure S3. Box plot graphs displaying normalized expression levels of GPR160 and NPY across major tissue groups divided into healthy and cancer samples in the Genesapiens transcriptomics database. [file 1471-2164-12-162-S7.PDF]

**A**

GPR160, ENSG00000173890

Healthy prostate

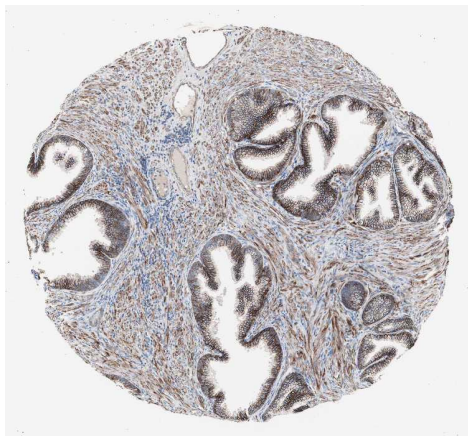

Prostate cancer

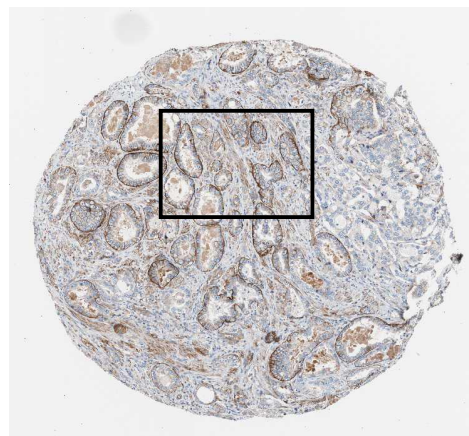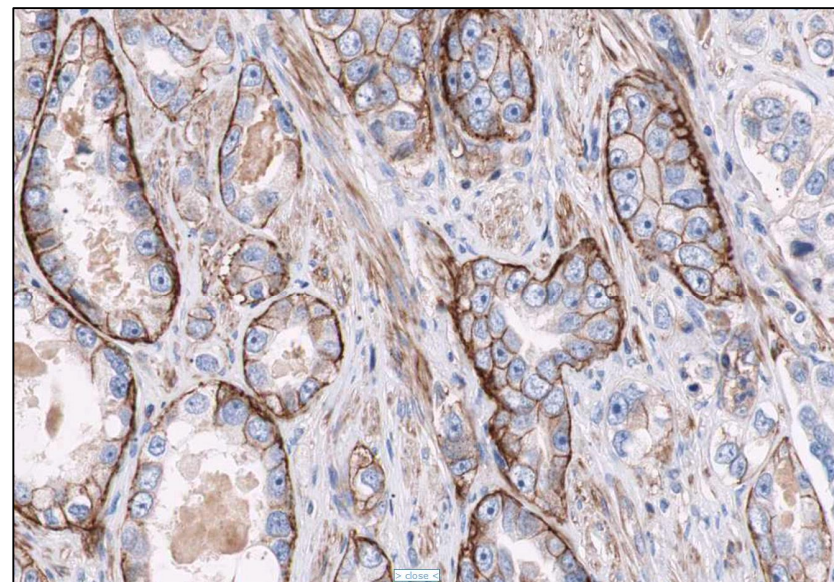**B**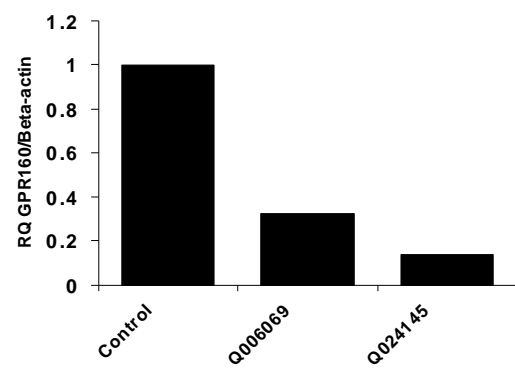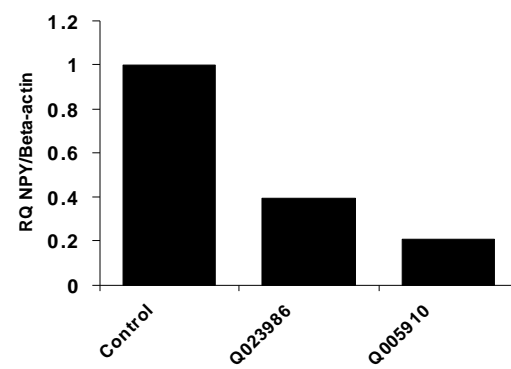

Supplement: Additional file 8 — Supplemental figure S4. (A) Representative immunohistochemistry staining of tissue microarray cores of healthy and prostate cancer samples for GPR160 http://www.proteinatlas.org. (B) Knockdown performance of siRNAs targeting GPR160 and NPY. Transcript levels for duplicate experiments with triplicate samples were measured by quantitative RT-PCR and are reported for each siRNA in the increasing efficacy order relative to average transcript levels for negative control siRNA transfections. [file 1471-2164-12-162-S8.PDF]
